# Association between metabolic score for insulin resistance and regression to normoglycemia from prediabetes in Chinese adults: A retrospective cohort study

**DOI:** 10.1371/journal.pone.0308343

**Published:** 2024-08-07

**Authors:** Xue-Hui Liu, Qiang Xu, Lei Zhang, Hong-Jun Liu

**Affiliations:** 1 Department of Cardiology, Yichang Hospital of Traditional Chinese Medicine, Yichang, China; 2 Three Gorges University Hospital of Traditional Chinese Medicine, Three Gorges University, Yichang, China; University of Montenegro-Faculty of Medicine, MONTENEGRO

## Abstract

**Background:**

Metabolic score for insulin resistance (METS-IR) is a surrogate index to estimate insulin sensitivity. The aim of this study was to examine the association between METS-IR and regression to normoglycemia in Chinese adults with prediabetes.

**Methods:**

A total of 15,415 Chinese adults with prediabetes defined by their fasting blood glucose were included in this retrospective study. The association between METS-IR and regression to normoglycemia from prediabetes was evaluated using the Cox proportional hazards regression model. A Cox proportional hazards regression with cubic spline function was performed to explore the nonlinear association between METS-IR and regression to normoglycemia. Kaplan-Meier curves was used to describe the probability of regression to normoglycemia from prediabetes.

**Results:**

In multivariate Cox proportional hazards regression analyses, the increase in METS-IR was independently associated with a reduced probability of regression to normoglycemia from prediabetes (all p < 0.01 in models 1–3). A nonlinear association between METS-IR and the probability of regression to normoglycemia was observed, with an inflection point of 49.3. The hazard ratio on the left side of the inflection point was 0.965 (95% CI 0.953–0.976). Subgroup analyses demonstrated the robustness of our findings.

**Conclusion:**

This study demonstrated a negative and nonlinear association between METS-IR and regression to normoglycemia in Chinese adults with prediabetes. When METS-IR is below 49.3, reducing METS-IR could significantly increase the probability of regression to normoglycemia from prediabetes.

## 1. Introduction

Prediabetes is a condition characterized by impaired fasting glucose (IFG), impaired glucose tolerance (IGT), or glycated hemoglobin A1c (HbA1c) levels between 5.7% and 6.4% [1]. The global prevalence of IFG and IGT in 2021 was 5.8% and 9.1% among adults aged 20–79 years. By 2045, the global prevalence of IFG and IGT is projected to increase to 6.5% and 10.0%, respectively [2]. According to the American Diabetes Association (ADA) expert panel, 70% of individuals with prediabetes will eventually progress to diabetes [3]. Prediabetes was associated with an increased risk of developing type 2 diabetes mellitus (T2DM) four- to fivefold compared with normoglycemia [4]. In addition, a meta-analysis of 129 prospective studies showed that prediabetes was associated with an increased risk of cardiovascular disease (CVD), cancer, and all-cause mortality over a median follow-up of 9.8 years [5]. Clinical trials have demonstrated that regression to normoglycemia is associated with a reduction in future diabetes and risk of CVD [6, 7]. Thus, screening for prediabetes and its risk factors, and restoring normoglycemia from prediabetes, is important.

Insulin resistance (IR) and defects in β-cell dysfunction are key factors in the progression from normoglycemia to prediabetes and to T2DM [8, 9]. Metabolic score for insulin resistance (METS-IR) is an index to assess cardiometabolic risk in healthy and at-risk individuals and a promising tool for screening of insulin sensitivity [10]. It has been demonstrated that METS-IR was better than triglyceride glucose (TyG) index and triglyceride to high-density lipoprotein cholesterol (TG/HDL-C) ratio in predicting the future T2DM [10]. Previous studies have shown a negative and nonlinear association between TyG index and TG/HDL-C ratio with regression to normoglycemia from prediabetes [11, 12]. However, the association between METS-IR and regression to normoglycemia has not been explored in individuals with prediabetes. In this study, we aimed to assess the association between METS-IR and the regression to normoglycemia among Chinese adults with prediabetes.

## 2. Methods

### 2.1 Study design

This study was a secondary analysis based on a cohort study performed by the China Rich Health Care Group, and the design has been described in a published study [13]. The data used in this study were obtained from a free public database (www.datadryad.org), which allowed researchers to download and use original data. Hence, there was no requirement for informed consent or approval from the participants. The reporting of the study was according to the STROBE statement [14]. The dataset was extracted from a previous article [13] titled “Association of body mass index and age with incident diabetes in Chinese adults: a population-based cohort study” (http://dx.doi.org/10.1136/bmjopen-2018-021768).

The independent variable was the METS-IR, which was defined as following: Ln(2×FPG(mg/dL)+TG(mg/dL))×BMI(kg/m^2^)/Ln(HDL-C(mg/dL)) (FPG: fasting plasma glucose, TG: triglyceride, BMI: body mass index, HDL-C: high-density lipoprotein cholesterol) [10]. METS-IR was calculated at baseline. The dependent variable was the regression to normoglycemia from prediabetes during the follow-up.

### 2.2 Participants

The China Rich Healthcare Group Cohort Study was established between 2010 and 2016, recruited 685,277 Chinese adults from 32 regions and 11 cities. According to exclusion criteria, 473,444 participants were excluded. The exclusion criteria was as follows [13]: (1) participants lacking baseline information on weight, height, gender, and FPG values; (2) extreme BMI values (<15 kg/m^2^ or >55 kg/m^2^); (3) participants with visit intervals less than two years; (4) participants with diabetes at baseline; (5) participants with undefined diabetes status at follow-up.

**Fig 1:**
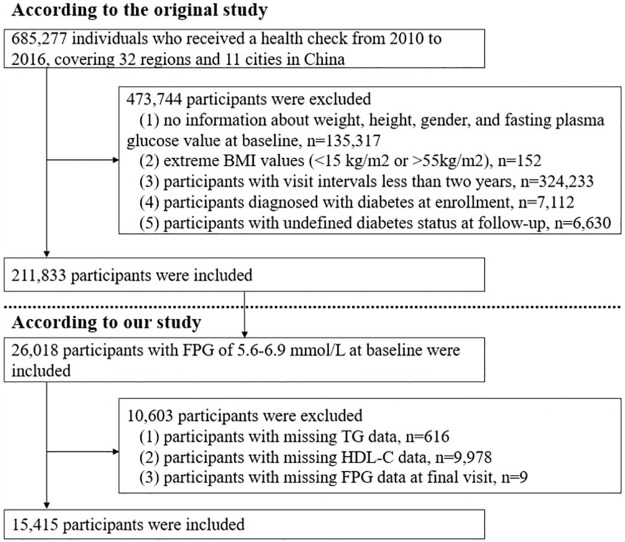
Flowchart of study participants. BMI, body mass index; FPG, fasting plasma glucose; HDL-C, high-density lipoprotein cholesterol; TG, triglyceride.

In this study, we extracted 26,018 participants with a baseline FPG ranging from 5.6 to 6.9 mmol/L, in accordance with the diagnostic criteria of the 2021 ADA guideline. Additionally, we excluded participants with no baseline information on TG and HDL-C, as well as those with no final FPG values. Finally, a total of 15,415 participants were included in this study. The procedure for participant selection was presented in Fig 1.

### 2.3 Data collection

In the original study, questionnaires were used to collect information on demographic, lifestyle, medical history, and family history of chronic diseases [13]. Height, weight and blood pressure were measured by trained staff. Blood pressure was measured by standard mercury sphygmomanometers. Weight was measured in light clothing with no shoes. Fasting venous blood samples were collected after fasting for ≥ 10 hours and measured by Beckman 5800 biochemical analyzer [13].

The selection of variables for this study was based on the original study. The covariates included gender, age, BMI, FPG, systolic blood pressure (SBP), diastolic blood pressure (DBP), TG, total cholesterol (TC), HDL-C, low-density lipoprotein cholesterol (LDL-C), alanine aminotransferase (ALT), aspartate aminotransferase (AST), serum creatinine (Scr), family history of diabetes, smoking status, and alcohol consumption status.

### 2.4 Statistical analysis

Participants were categorized into four groups based on the quartile of METS-IR value. Continuous variables were expressed as mean (standard deviation) or median (interquartile range), and comparisons between groups were assessed using one-way ANOVA test. Categorical variables were expressed as frequencies and percentages, and comparisons between groups were assessed using χ^2^ test. Univariate and multivariate Cox proportional hazards regression models were used to assess hazard ratios (HRs) and corresponding 95% confidence interval (CI) for the probability regression from prediabetes to normoglycemia. Kaplan-Meier survival curve was used for assessing the probability of regression to normoglycemia from prediabetes. We conducted the non-adjusted and two adjusted models in this study. Model 1 was a crude model with no adjusted covariates. Model 2 was adjusted for age and gender. Model 3 was adjusted for age, gender, SBP, DBP, TC, LDL-C, AST, ALT, Scr and family history of diabetes.

The Cox proportional hazards regression model with cubic spline functions was performed to explore the potential nonlinear relationship between METS-IR and the probability of regression to normoglycemia from prediabetes. A two-piecewise linear regression model was performed to determine the threshold effect.

Subgroup analyses were conducted on different subgroups (age, gender, BMI, SBP, and DBP) using stratified Cox proportional hazards regression models.

Statistical analyses were performed using SPSS 19.0 (SPSS Inc., Chicago, Illinois, USA) and RStudio (http://github.com/rstudio/rstudio). A two-sided P value < 0.05 was considered statistical significance.

## 3. Results

### 3.1 Characteristics of study participants

Table 1 presented the characteristics of participants with prediabetes. A total of 15,415 participants (10,003 male and 5,412 female) with prediabetes at baseline were included. The mean age was 50.9 years. The median follow-up duration was 3.0 years. A total of 6,682 participants experienced a regression to normoglycemia from prediabetes. The overall rate of regression to normoglycemia was 144.5 per 1,000 person-years in individuals with prediabetes. The METS-IR was a skewed fractional distribution, with a median of 37.176. The participants were classified into subgroups based on the quartiles of METS-IR (Q1: ≤32.865, Q2: 32.866–37.176, Q3: 37.177–41.726, Q4: ≥41.727). Compared with participants in the Q1 group, those in the Q4 group had significant increases in FPG, SBP, DBP, BMI, TG, TC, and creatinine, while there was an opposite trend in HDL-C. Participants in the Q1 group had higher incidence rates of glucose status conversion when compared with Q4 group (56.8% vs 34.2%, P < 0.001).

**Table 1:** Baseline characteristics of participants.

Characteristic	METS-IR				P value
	Q1 (≤32.865)	Q2 (32.866–37.176)	Q3 (37.177–41.726)	Q4 (≥41.727)	
N	3,853	3,854	3,852	3,856	
Age, year	48.31±14.28	51.93±13.35	52.42±12.88	51.03±12.82	< 0.001
Male, n (%)	1716 (44.5)	2433 (63.1)	2744 (71.2)	3110 (80.7)	< 0.001
BMI, kg/m2	21.06±1.65	23.92±1.34	25.78±1.60	28.58±2.63	< 0.001
FPG, mmol/L	5.88±0.27	5.94±0.31	5.98±0.33	6.02±0.34	< 0.001
TG, mmol/L	1.04±0.51	1.46±0.78	1.91±1.13	2.8±2.02	< 0.001
TC, mmol/L	4.94±0.94	5.03±0.93	5.06±0.92	5.14±0.98	< 0.001
HDL-C, mmol/L	1.55±0.32	1.38±0.24	1.28±0.23	1.13±0.25	< 0.001
LDL-C, mmol/L	2.87±0.71	2.97±0.71	2.96±0.70	2.93±0.76	< 0.001
SBP, mmHg	121.86±17.46	127.20±17.31	129.13±17.45	131.81±17.04	< 0.001
DBP, mmHg	74.43±10.50	77.67±10.59	79.73±10.96	82.20±11.19	< 0.001
ALT	19.10±14.01	24.35±17.63	30.34±26.23	38.85±27.06	< 0.001
AST	23.28±9.69	24.80±10.97	26.39±9.39	29.77±13.69	< 0.001
Scr, mmol/L	68.05±15.41	73.08±16.53	74.81±15.47	76.17±16.08	< 0.001
Smoking status, n (%)					< 0.001
Current	161 (4.2)	247 (6.4)	337 (8.7)	467 (12.1)	
Past	41 (1.1)	59 (1.5)	56 (1.5)	68 (1.8)	
Never	935 (24.3)	813 (21.1)	835 (21.7)	723 (18.8)	
Not recorded	2716 (70.5)	2735 (71.0)	2624 (68.1)	2598 (67.4)	
Drinking status, n (%)					< 0.001
Current	25 (0.6)	48 (1.2)	71 (1.8)	83 (2.2)	
Past	155 (4.0)	246 (6.4)	262 (6.8)	278 (7.2)	
Never	957 (24.8)	825 (21.4)	895 (23.2)	897 (23.3)	
Not recorded	2716 (70.5)	2735 (71.0)	2624 (68.1)	2598 (67.4)	
Family history of DM, n (%)					> 0.05
Yes	91 (2.4)	111 (2.9)	98 (2.5)	100 (2.6)	
No	3762 (97.6)	3743 (97.1)	3754 (97.5)	3756 (97.4)	

ALT, alanine aminotransferase; AST, aspartate aminotransferase; BMI, body mass index; DBP, diastolic blood pressure; DM, diabetes mellitus; FPG, fasting plasma glucose; HDL-C, high-density lipoprotein cholesterol; LDL-C, low-density lipoprotein cholesterol; METS-IR, metabolic score for insulin resistance; SBP, systolic blood pressure; Scr, serum creatinine; TC, total cholesterol; TG, triglyceride.

### 3.2 Univariate analyses using Cox proportional hazards regression model

The result of the univariate Cox proportional hazards regression analyses was presented in Table 2. Negative associations were found between regression to normoglycemia and age, BMI, SBP, DBP, FPG, LDL-C, TG, ALT, AST, and Scr, while a positive association was noted with HDL-C.

**Table 2:** Univariate Cox proportional hazards regression was used to assess the confounding factors influencing the probability of regression to normoglycemia from prediabetes.

Confounding factor	HR (95%CI)	P
METS-IR	0.958 (0.954, 0.962)	< 0.001
Age	0.974 (0.972, 0.976)	< 0.001
Sex		
Male	1	
Female	1.250 (1.190, 1.313)	< 0.001
BMI	0.934 (0.927, 0.941)	< 0.001
SBP	0.988 (0.987, 0.990)	< 0.001
DBP	0.984 (0.982, 0.986)	< 0.001
FPG	0.217 (0.197, 0.240)	< 0.001
TC	0.887 (0.864, 0.910)	< 0.001
HDL-C	1.908 (1.786, 2.037)	< 0.001
LDL-C	0.937 (0.906, 0.969)	< 0.001
TG	0.902 (0.883, 0.921)	< 0.001
ALT	0.994 (0.993, 0.995)	< 0.001
AST	0.992 (0.988, 0.996)	< 0.001
Scr	0.997 (0.995, 0.998)	< 0.001
Family history of diabetes		
No	1	
Yes	1.211 (1.040, 1.410)	0.013

ALT, alanine aminotransferase; AST, aspartate aminotransferase; BMI, body mass index; DBP, diastolic blood pressure; FPG, fasting plasma glucose; HDL-C, high-density lipoprotein cholesterol; LDL-C, low-density lipoprotein cholesterol; METS-IR, metabolic score for insulin resistance; SBP, systolic blood pressure; Scr, serum creatinine; TC, total cholesterol; TG, triglyceride.

**Fig 2:**
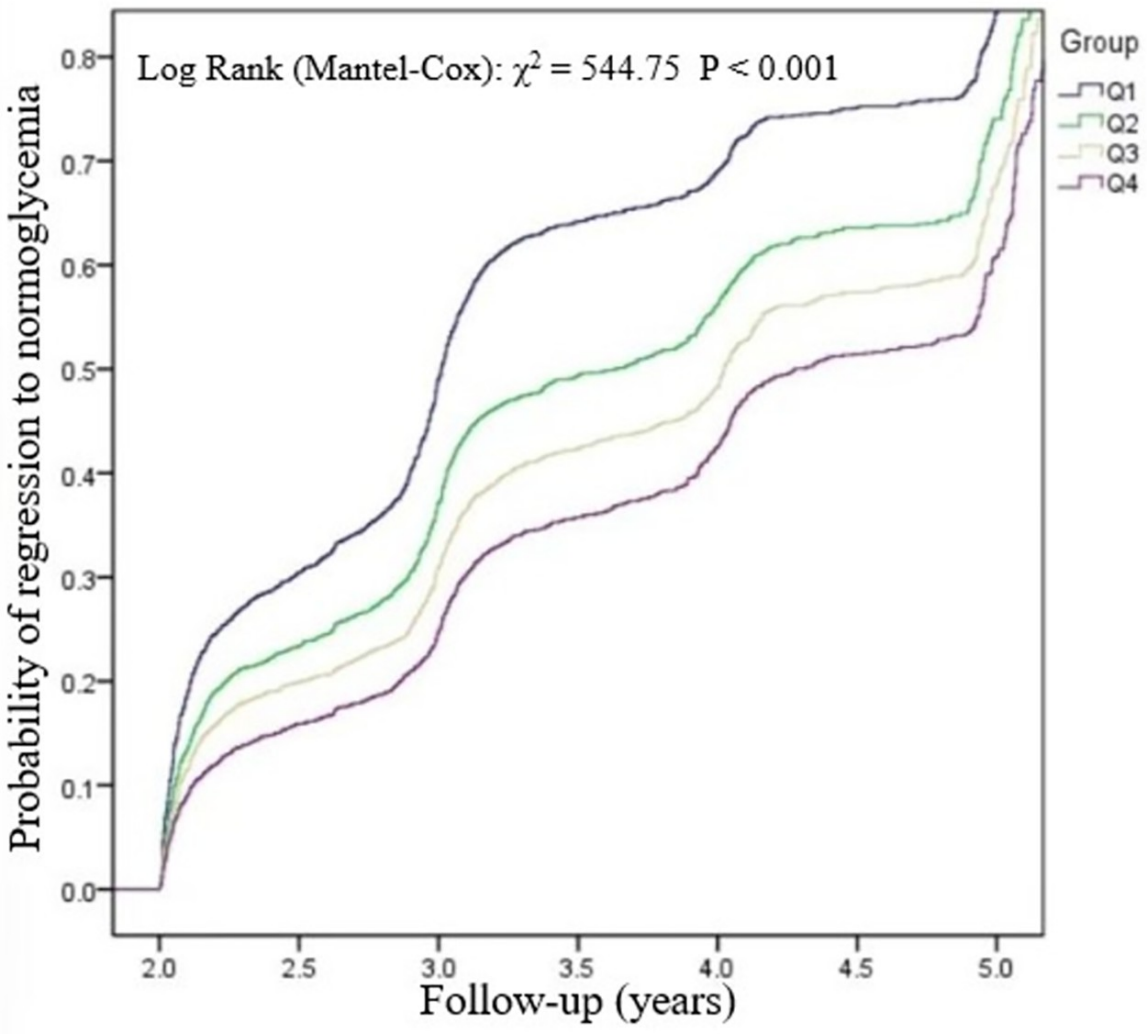
Kaplan-Meier curves for the probability of regression to normoglycemia from prediabetes.

Kaplan-Meier curves illustrating normoglycemia survival probability stratified by METS-IR group were shown in Fig 2. Significant differences in the probability of regression to normoglycemia from prediabetes among METS-IR groups were observed (log-rank test, p<0.001). The rate of regression to normoglycemia decreased as METS-IR increased, indicating that individuals with the highest METS-IR had the lowest probability of regression to normoglycemia.

### 3.3 Multivariate analyses using Cox proportional hazards regression model

Three Cox proportional hazards regression models were predefined to evaluate the association between METS-IR and the regression to normoglycemia (Table 3). In the crude model, a 1-unit increase in METS-IR was linked to a 4.2% reduction in the likelihood of regression to normoglycemia. In the model 2, after adjusting for age and gender, each unit increase of METS-IR was associated with a 3.5% decrease in the probability of regression to normoglycemia. In the model 3, the HR was 0.967 (95% CI 0.960–0.973), showing a significant statistical difference.

**Table 3:** Multivariate Cox proportional hazards regression was used to assess the confounding factors influencing the probability of regression to normoglycemia from prediabetes.

Exposure	Model 1 (HR, 95% CI, P)	Model 2 (HR, 95% CI, P)	Model 3 (HR, 95% CI, P)
METS-IR	0.958 (0.954, 0.962) P<0.01	0.965 (0.961, 0.968) P<0.01	0.967 (0.960, 0.973) P<0.01
METS-IR groups			
Q1	1.0	1.0	1.0
Q2	0.709 (0.665, 0.755) P<0.01	0.789 (0.739, 0.841) P<0.01	0.827 (0.747, 0.915) P<0.01
Q3	0.582 (0.544, 0.621) P<0.01	0.667 (0.623, 0.715) P<0.01	0.655 (0.587, 0.731) P<0.01
Q4	0.474 (0.443, 0.508) P<0.01	0.529 (0.493, 0.569) P<0.01	0.570 (0.505, 0.642) P<0.01)
P for trend	P<0.01	P<0.01	P<0.01

Model 1 was a crude model with no adjusted covariates,

Model 2 was adjusted for age and gender.

Model 3 was adjusted for age, gender, blood pressure, total cholesterol, low-density lipoprotein cholesterol, alanine aminotransferase, aspartate aminotransferase, serum creatinine, and family history of diabetes.

When METS-IR was treated as a categorical variable and analyzed in the fully adjusted model. Comparing with participants in the first quartile of METS-IR, the HR in the second quartile was 0.827, indicating a 17.3% lower probability of regression to normoglycemia. For participants in the fourth quartile, the HR was 0.570, suggesting a 43.0% lower probability of regression to normoglycemia.

### 3.4 Assessing nonlinear association in the Cox proportional hazards regression model using cubic spline functions

**Fig 3:**
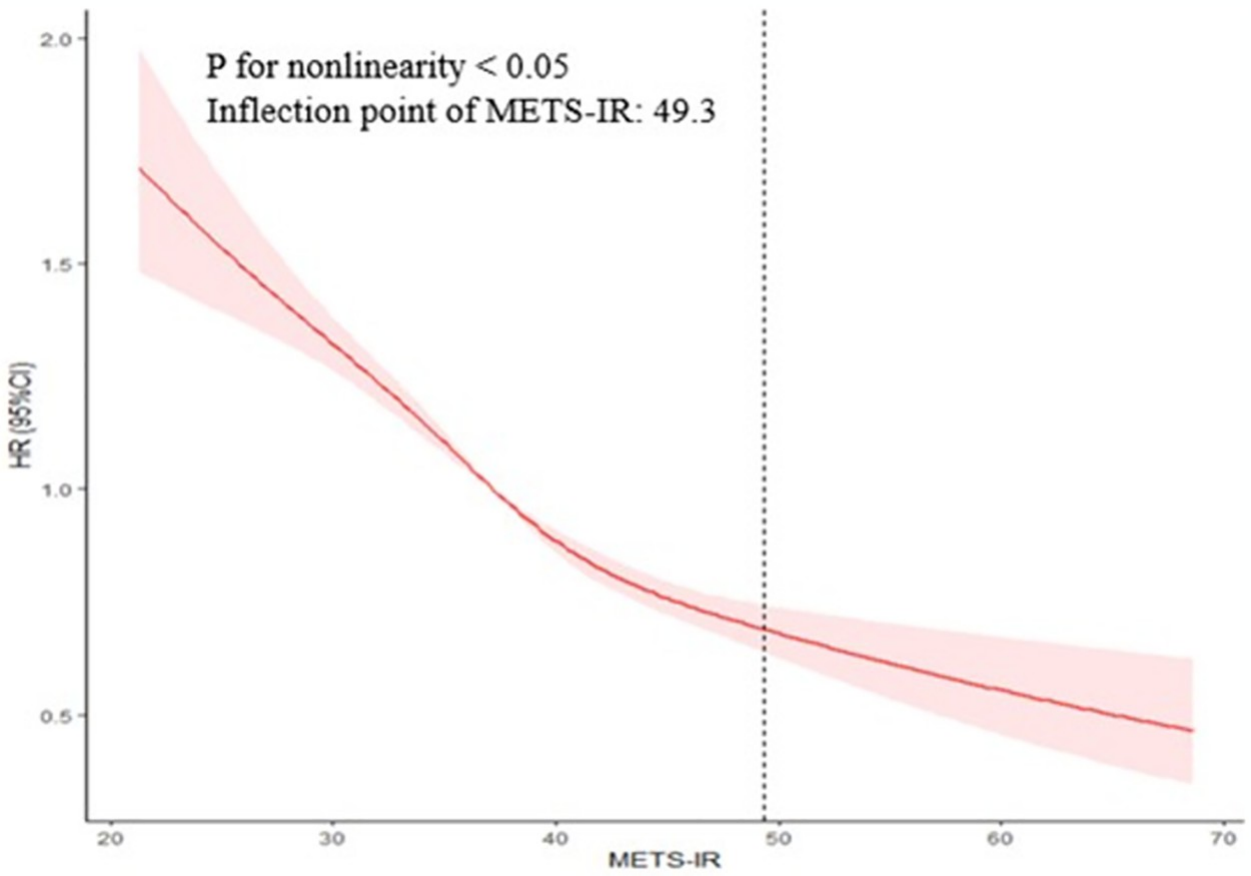
The nonlinear association between METS-IR and the probability of regression to normoglycemia in individuals with prediabetes. METS-IR, metabolic score for insulin resistance.

A nonlinear association was observed between METS-IR and regression to normoglycemia in the Cox proportional hazards regression model using cubic spline functions analyses (Fig 3). The inflection point of curve was identified at 49.3 using two-piecewise linear regression. On the left side of the inflection point, the HR was 0.964 (95%CI, 0.960–0.968). On the right of the inflection point, the HR was 1.008 (95%CI, 0.986–1.030), with no significant difference observed.

### 3.5 Subgroup analysis

**Fig 4:**
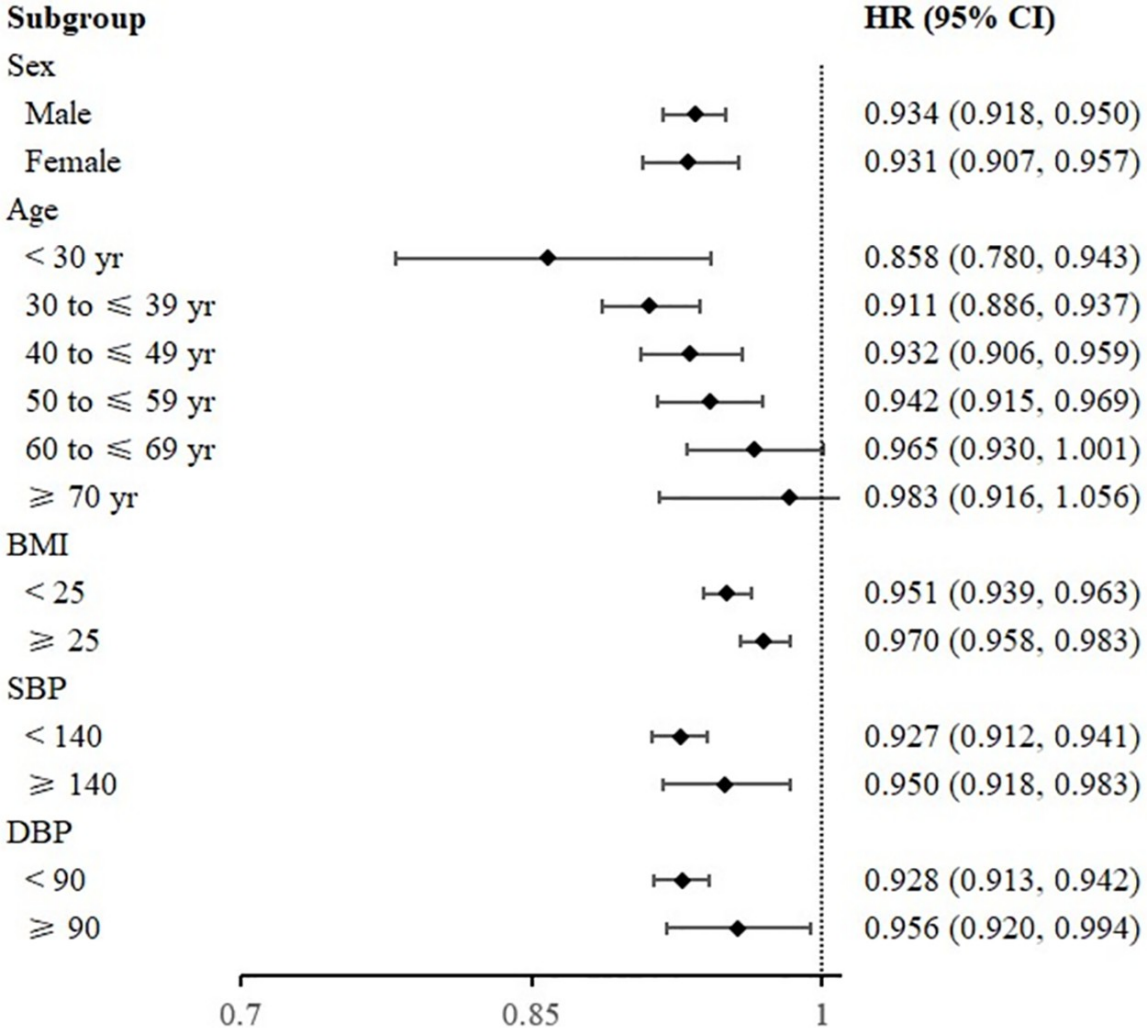
Effect of metabolic score for insulin resistance on the regression to normoglycemia from prediabetes in specified subgroups. BMI, body mass index; DBP, diastolic blood pressure; SBP, systolic blood pressure.

No substantial interaction was observed between METS-IR and the rate of regression to normoglycemia based on gender, age, BMI, SBP, and DBP stratification (Fig 4). It is noteworthy that the predictive ability of METS-IR is greater for individuals aged < 60 years.

## 4. Discussion

In this study, we found that 43.34% of participants reverted to normoglycemia over a median follow-up of 3.0 years. There was a negative and nonlinear association between METS-IR and regression to normoglycemia in Chinese adults with prediabetes. The inflection point of the curve was 49.3. When the METS-IR less than 49.3, the probability of regression to normoglycemia decreased as METS-IR values increased.

Prediabetes affected approximately 720 million individuals worldwide in 2021 and will affect an estimated 1 billion individuals by 2045 [2]. Prediabetes is not a single entity but a heterogeneous condition diagnosed by different definitions, with varying prevalence, response to lifestyle interventions, probability of regression to normoglycemia, risk of progression to T2DM, and risk of developing long-term complications [15]. The major risk factors for prediabetes included overweight or obesity, unhealthy diet, physical inactivity, older age and genetic predisposition [16]. Individuals with prediabetes have an increased risk of T2DM, CVD, cancer and all-cause mortality [4, 5]. A meta-analysis of 103 prospective cohort studies showed that prediabetes was associated with an increased risk of developing T2DM four- to fivefold compared with normoglycemia. Another meta-analysis of 129 prospective cohort studies demonstrated that prediabetes was associated with an increased risk of CVD, coronary heart disease, stroke, and all-cause mortality over a median follow-up of 9.8 years.

Clinical trials have shown that individuals with prediabetes (those with IGT) can reduce their risk for T2DM through lifestyle modification or drug therapies [17]. Lifestyle modification is considered as the first-line therapy for prediabetes, without major adverse events [16]. In most randomized controlled trials, the effects of lifestyle modification were primarily mediated by loss of weight. The ADA recommends at least 7% weight loss through healthy diet and approximately 150 min/week of moderate-intensity physical activity in individuals with prediabetes [1]. These approaches can prevent or delay disease progression and even regress to normoglycemia in individuals with prediabetes. In addition, previous studies have demonstrated that even a temporary reversion to normoglycemia is associated with a reduction in the future risk of T2DM and its associated complications among individuals with prediabetes [6, 7]. Thus, regression to normoglycemia from prediabetes should not be ignored.

Previous studies have shown that a substantial proportion of European populations with prediabetes regress to normoglycemia, and regression rates are often higher than the progression rates to T2DM [15]. A meta-analysis of 35 randomized controlled trials including 10,164 adults with prediabetes showed that 31% of control participants regressed to normoglycemia over a median follow-up of 1.6 years [18]. In a Chinese prospective cohort study of 14,231 prediabetes (aged ≥18 years), after a mean follow-up of 2 years, 44.92% regressed to normoglycemia as opposed to only 13.29% developing T2DM [6]. It similar to our study, 43.34% of participants regressed to normoglycemia over a median follow-up of 3.0 years. Therefore, identifying the triggers and predictors of regression to normoglycemia from prediabetes is important for the prevention of developing T2DM and long-term complications.

IR is a risk factor for the development of dyslipidemia, hypertension, CVD, and T2DM. The surrogate indices of IR, such as the TyG index, TG/HDL ratio, and TyG-BMI, have all been assessed for their ability to predict the probability of regression to normoglycemia from prediabetes [11, 12, 19]. Shao Y et al found that there is a negative and nonlinear relationship between TyG-BMI and regression to normoglycemia in Chinese adults with prediabetes [19]. When TyG-BMI was ≥ 196.4, it was significantly and negatively associated with the probability of regression to normoglycemia. There are also negative and nonlinear associations between TG/HDL ratio, TyG index and regression to normoglycemia in prediabetes [11, 12]. METS-IR, a surrogate index to estimate insulin sensitivity, is calculated using FPG, TG and HDL-C along with BMI, which are routinely obtained and do not rely on fasting insulin measurements [10]. METS-IR exhibited significant correlations with visceral, intrahepatic and intrapancreatic fat, which are recognized as pathophysiological components of IR [10]. Assessing at-risk individuals with METS-IR could identify pathophysiological changes in IR, avoiding the expense and variability of fasting insulin tests. Recently, METS-IR has been proposed as a more promising and reliable index for assessing IR [10]. Clinical studies have demonstrated a strong association between METS-IR and hypertension, T2DM, and CVD [20–23]. The mechanisms for these associations may be related to IR.

Our findings indiciate a negative and nonlinear association between METS-IR and regression to normoglycemia in Chinese adults with prediabetes. A 1-unit increase in METS-IR was linked to a 3.3% decrease in the probability of regression to normoglycemia. The mechanisms responsible for the negative association between METS-IR and regression to normoglycemia in individuals with prediabetes remain unclear, but they could be related to IR. Previous studies have reported the role of IR in both the progression and regression of prediabetes [24, 25]. The inflection point of METS-IR was 49.3. Below 49.3, the probability of regressing to normoglycemia decreased as METS-IR levels rose. However, above 49.3, no significant association was observed. This finding provided METS-IR as a simple and low-cost stratification tool to assess the likelihood of regressing to normoglycemia form prediabetes in Chinese adults. Moreover, it aids primary care physicians in decision-making processes to optimize diabetes prevention strategies in individuals with prediabetes.

Subgroup analysis presents that age influences the association between METS-IR and regression to normoglycemia. Individuals aged < 60 years showed a notable inverse correlation between METS-IR and regression to normoglycemia compared to those aged ≥ 60 years. For the relatively younger individuals, METS-IR demonstrates greater predictive ability for assessing regression to normoglycemia from prediabetes, possibly linked to β-cell function. T2DM is an age-related disease characterized by a decrease of β-cell function. In a large prospective study, Liu J *et al* found that aging is associated with decreased β-cell function and increased IR in Chinese population [26]. Individuals who regressed to normoglycemia from prediabetes showed a significant increase in β-cell levels during the 3 years of follow-up. Therefore, maintenance of normal glucose regulation mainly depends on the ability to compensatory increase of the β-cell function.

### 4.1 Strengths and limitations

Our study’s strength lies in the comprehensive evaluation of the association between METS-IR and regression to normoglycemia from prediabetes using a large sample of the Chinese population. Therefore, the findings of this study are likely to have broad relevance for Chinese adults. However, the study had some limitations. First, the definition of prediabetes and regression to normoglycemia did not include measurements of a 2-hour oral glucose tolerance test or HbA1c levels, which could potentially lead to underestimation or overestimation of the likelihood of regression to normoglycemia from prediabetes. Second, a significant number of participants had missing data on drinking and smoking status. Consequently, we excluded these two variables during the Cox proportional hazards regression analysis, which could impact statistical power due to missing data. Thirdly, despite adjusting for a wide range of confounding factors, residual confounding resulting from measurement errors in the assessment cannot be ruled out [13]. Finally, this study involved a secondary analysis of previously published retrospective data, limiting the ability to establish a causal association.

## 5. Conclusion

This study demonstrated a negative and nonlinear association between METS-IR and regression to normoglycemia in Chinese adults with prediabetes. When the METS-IR was < 49.3, it was negatively associated with the probability of regression to normoglycemia from prediabetes. This data provides crucial clinical insights to support early and aggressive interventions for regression to normoglycemia in prediabetes with METS-IR < 49.3.
